# Grand challenges in stem cell treatments

**DOI:** 10.3389/fcell.2013.00002

**Published:** 2013-10-10

**Authors:** Susumu Ikehara

**Affiliations:** Department of Stem Cell Disorders, Kansai Medical UniversityHirakata City, Osaka, Japan

**Keywords:** stem cell transplantation, immunorejection, ESC, IPS, tissue stem cell

Stem cells mainly include embryonic stem cells (ESC), pluripotent stem cells (iPS), Epiblast-derived stem cells (Epi-SC), and adult tissue stem cells. ESC are pluripotent, being able to differentiate into the ectoderm, mesoderm and endoderm (Evans and Kaufman, 1981; Martin, 1981). However, there are ethical issues when using human embryos, and ESC transplants into mouse models can result in malignant tumors. Further challenges facing research in the use of ESC are how to reduce contamination, and prevent cancer risk. Human ESC lines derived from single blastomeres have been reported (Klimanskaya et al., 2006). This method may create new stem cell lines and therapies without destroying embryos, and would thus address the ethical concerns and allow the generation of matched tissues for children and siblings born from transferred preimplantation genetic diagnosis embryos. Another current challenge is that the cells used in research are rejected by the recipient's immune system. Yamanaka et al. reported the induction of iPS from mouse embryonic and adult fibroblast cultures by introducing four factors: Oct3/4, Sox2, c-Myc, and Klf4 (Takahashi and Yamanaka, 2006). Inducing pluripotent cells directly from the patient's own cells to generate tissue or cells may resolve some of the problems associated with using ESC, including the problems of immunorejection and the ethical issues. However, the low frequency of iPS cell derivation is still unresolved. A major difficulty that scientists continue to encounter is the identification of stem cells in adult tissues. EpiSCs are derived from post-implantation mouse embryos from embryonic day 5.5 to E7.5 (Brons et al., 2007). They show pluripotency and require Nodal-Activin and fibroblast growth factor signaling for maintenance of this characteristic. Therefore, they are considered to be more closely related to human ESC than to mouse ESC (Tesar et al., 2007). Adult stem cells such as bone marrow stem cells, intestinal stem cells (Van Der Flier and Clevers, 2009), endothelial stem cells (Rafii, 2000), and neural stem cells (Lewis, 1968) have been detected in tissues. One challenge facing researchers is how to control and regulate the process to successfully trigger differentiation into the desired cell type after the stem cells have been isolated. When some defined transcription factors are transferred into somatic cells, somatic cells can directly reprogram to differentiate into other cell types without passing through a pluripotent state. Some reports demonstrated that mouse fibroblasts can directly reprogram to neural stem cells (Han et al., 2012), cardiomyocytes (Ieda et al., 2010), bipotencial hepatic stem cells (Yu et al., 2013), and endothelial cells (Li et al., 2013). However, the restricted proliferative and lineage potential of the resulting cells limits the scope of their potential applications.

Stem cell treatments include new technologies and therapies that aim to replace damaged tissues and cells in order to treat disease or injury (Lindvall and Kokaia, 2006). Stem cells have the power to congregate in these damaged areas and generate new cells and tissues by performing a repair and renewal process, restoring functionality. ESC, iPS, and adult stem cell therapies, which include bone marrow stem cells and peripheral stem cells are currently being investigated or used to treat a range of diseases. Bone marrow stem cells are used to replace blood cells in people suffering from leukemia and other cancers (Goldman et al., 1988). Burn victims are also benefiting from stem cell therapy, which allows for new skin cells to be grafted as a replacement for those that have been damaged (Janes et al., 2002).

The ability of stem cells to self-renew and their capacity for differentiation offers significant potential for the generation of tissues with minimal risk of rejection and side effects. A number of stem cell therapies are still in the experimental stages, and are controversial (Francis et al., 2013). The first challenge researchers face when considering stem cell treatment is to understand the mechanisms by which stem cells function in the injured microenvironment using animal models, and then to translate the results of these studies to humans. Another challenge is how to identify and isolate stem cells from tissue, and then induce their differentiation into the desired cell types. The third challenge is how to prevent immunorejection after stem cell transplantation. Immunological rejection is a major barrier to successful stem cell transplantation. A person's immune system may also recognize the transplanted cells as foreign bodies and this can trigger an immune reaction that results in the rejection of the transplanted cells. Recipients of the transplant usually have to take strong immunosuppressive drugs to reduce the chances of rejection but these drugs induce infection by viruses or microbes in the environment (van Sandwijk et al., 2013).

The goal of *Stem Cell Treatments* is to publish all findings and experiences of stem cell treatments, experimentally and clinically, and to open up new possibilities for the treatment of various diseases such as Alzheimer's disease, traumatic brain injury, heart disease, diabetes, and cancer, etc., that we may promote health and extend the human life span.
